# RNA m^6^A Modification in Immunometabolism: Context-Dependent Mechanisms and Therapeutic Perspectives

**DOI:** 10.3390/ijms27219566

**Published:** 2026-10-26

**Authors:** Qiong Fang, Wei Li, Yongqing Cai, Shuyi Zhang, Xinzhi Tang, Chu Chu, Miaomiao Zhou, Fang Li, Zhen Zhang, Xia Li

**Affiliations:** 1 Innovative Institute of Chinese Medicine and Pharmacy, Shandong University of Traditional Chinese Medicine, Jinan 250355, China; sdzyydxfq@163.com (Q.F.); li13793673390@163.com (W.L.); sdcyq725@163.com (Y.C.); tangxinzhia@163.com (X.T.); 61010216@sdutcm.edu.cn (C.C.); 60230097@sdutcm.edu.cn (M.Z.); 60230129@sdutcm.edu.cn (F.L.); 2 Jinan Key Laboratory of Traditional Chinese Medicine Immunoregulation, Traditional Chinese Medicine Immunoregulation Engineering Research Center of Shandong Province, Key Laboratory of Integrative Innovation in Traditional Chinese Medicine and Immunology of Shandong Province, Key Laboratory of Traditional Chinese Medicine Classical Theory, Ministry of Education, Shandong University of Traditional Chinese Medicine, Jinan 250355, China; zsy780921@163.com; 3 College of Traditional Chinese Medicine, Shandong University of Traditional Chinese Medicine, Jinan 250355, China

**Keywords:** N^6^-methyladenosine, metabolic reprogramming, immune cells, epitranscriptomics, pharmacological targeting

## Abstract

N^6^-methyladenosine (m^6^A) regulates RNA fate and has been linked to immune cell function and metabolic adaptation. This narrative review examines selected studies across innate, adaptive, and tissue-resident immune populations, focusing on the cellular origin, target transcript, and experimental evidence supporting each mechanism. Metabolic inputs, including α-ketoglutarate, lactate, and D-2-hydroxyglutarate, can influence m^6^A regulatory pathways, while m^6^A-dependent RNA regulation can alter metabolic enzymes, transporters, and immune responses. We discuss these findings within a proposed metabolite–m^6^A–immune framework; reciprocal relationships demonstrated in separate settings do not establish a closed feedback circuit in every cell population or disease model. We distinguish direct metabolic measurements from changes in signaling, survival, differentiation, and inferred pathway activity, and consider how tissue context and target selection may account for contrasting effects of the same regulator. Representative pharmacological studies of METTL3, FTO, and YTH domain readers are considered alongside interventions with less well-defined target specificity and early clinical development of METTL3 inhibition. The available evidence supports context-dependent mechanisms and therapeutic hypotheses, while clinical benefit, cellular selectivity, and long-term safety remain unresolved for the applications discussed.

## 1. Introduction

N^6^-methyladenosine (m^6^A) is the most prevalent internal modification in eukaryotic messenger RNA. The identification of FTO as an m^6^A demethylase by Jia et al. in 2011 established that this modification can be dynamically reversed [1]. Its deposition, removal, and recognition are mediated by methyltransferases (writers), demethylases (erasers), and m^6^A-binding proteins (readers). METTL3-METTL14 constitutes the major writer complex, FTO and ALKBH5 act as demethylases, and YTH domain proteins are prominent readers [2,3]. By influencing RNA processing, stability, translation, and degradation, these regulators shape gene expression programs in physiological and pathological settings [3,4].

Cellular metabolism provides not only the energetic foundation for immune cell survival but also serves as a determinant of immune cell identity and function. The concept of metabolic reprogramming, initially described in cancer biology, has been increasingly recognized as a fundamental mechanism controlling immune cell activation, differentiation, and effector responses [5,6,7]. Depending on their activation state and microenvironmental signals, immune cells undergo distinct metabolic transitions. Resting immune cells predominantly rely on oxidative phosphorylation (OXPHOS) and fatty acid oxidation (FAO) to maintain cellular homeostasis, whereas activated immune cells frequently shift toward aerobic glycolysis to rapidly generate energy and biosynthetic intermediates required for immune responses [8]. Such metabolic plasticity enables immune cells to adapt to changing physiological and pathological conditions; however, dysregulated metabolic remodeling may lead to immune dysfunction and contribute to the development of cancer, autoimmune disorders, and metabolic diseases [9,10].

Increasing evidence indicates that immune metabolic dysregulation represents a central feature of multiple human diseases. Within the tumor microenvironment (TME), metabolic competition, nutrient depletion, and accumulation of inhibitory metabolites drive immune cell exhaustion and facilitate immune escape [11]. In obesity and type 2 diabetes, chronic inflammatory states alter immune cell metabolic programs and contribute to impaired insulin sensitivity [12,13]. Similarly, autoimmune diseases such as rheumatoid arthritis and multiple sclerosis are characterized by excessive glycolytic activity and metabolic alterations in activated T cells and macrophages [14,15]. In cardiovascular disorders, including atherosclerosis, metabolic imbalance within immune cells promotes inflammatory activation and disease progression [16,17]. These findings highlight that deciphering the regulatory mechanisms underlying immunometabolism is essential not only for understanding disease pathogenesis but also for identifying novel therapeutic strategies.

Importantly, accumulating evidence suggests that the relationship between m^6^A modification and cellular metabolism is bidirectional. On one hand, metabolic states and intracellular metabolites can influence m^6^A regulatory machinery. Metabolites involved in central metabolic pathways, including α-ketoglutarate (α-KG), lactate, D-2-hydroxyglutarate (D-2HG), and fatty acid derivatives, may regulate the activity or expression of m^6^A writers and erasers by affecting enzymatic activity, substrate availability, or signaling pathways. On the other hand, m^6^A modification regulates the expression and fate of transcripts encoding metabolic enzymes, transporters, signaling molecules, and immune regulators, thereby shaping immune cell metabolic adaptation and functional states [18,19]. These findings motivate a proposed metabolite–m^6^A–immune framework in which RNA regulation can both respond to and influence metabolism; they do not establish a shared closed feedback circuit across immune populations.

**Figure 1:**
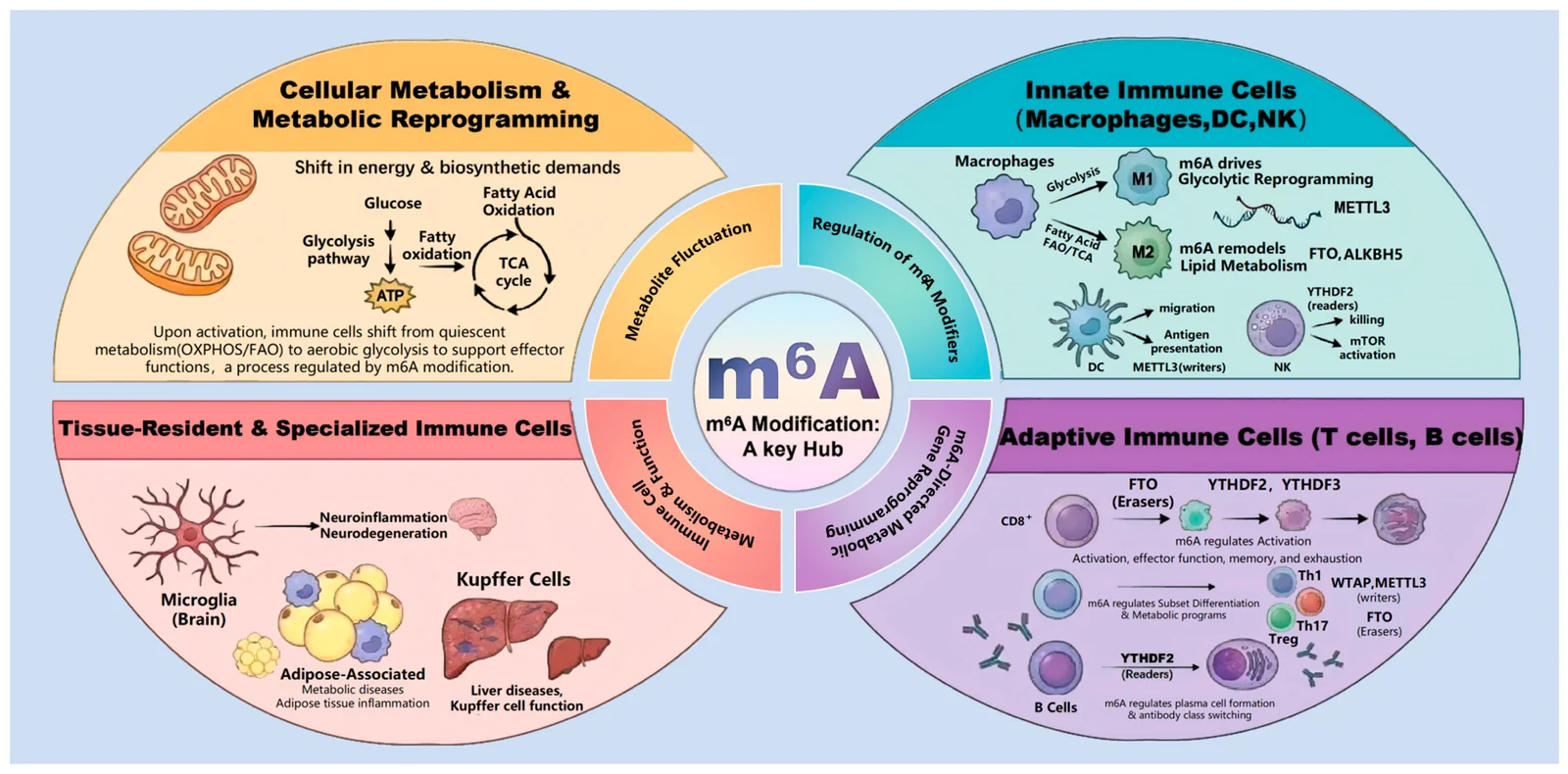
A proposed framework linking metabolic inputs, m^6^A-dependent RNA regulation, and immune function. The schematic integrates relationships reported in different cellular and disease settings; it does not imply that all connections constitute a validated closed feedback loop within a single model.

Recent reviews have examined m^6^A regulation in macrophages, immune cell development and function, B cell disease, and tissue-resident immune cell metabolism [20,21,22,23,24]. Here, we bring these perspectives together by examining the reciprocal relationship between metabolic signals and m^6^A-dependent RNA regulation across innate, adaptive, and tissue-resident immune populations (Figure 1). The comparison focuses on which metabolic input changes an m^6^A regulator, which transcript is affected, and how the resulting response depends on the cellular setting.

This approach helps explain why the same regulator can support immune protection in one setting and contribute to disease in another. We compare the effects of FTO and other regulators in relation to cell identity, metabolic environment, and target transcript, while distinguishing experimentally supported mechanisms from proposed connections. These comparisons also inform the therapeutic discussion: an intervention that benefits one cell population may impair another, making cellular selectivity as important as the choice of molecular target.

### Literature Search Strategy

This narrative review was based on literature searches conducted in PubMed, Web of Science Core Collection, and Scopus using combinations of controlled vocabulary and free-text terms related to “m^6^A”, “N^6^-methyladenosine”, “RNA epitranscriptomics”, “immunometabolism”, “metabolic reprogramming”, “immune cells”, “inflammation”, “cancer”, and “autoimmune disease”. We prioritized peer-reviewed original research articles, mechanistic studies, pharmacological studies, translational studies, and authoritative reviews focusing on m^6^A writers, erasers, readers, metabolites, metabolic regulation, and immune cell functions. Relevant studies were selected based on their contributions to understanding the interactions between m^6^A modification, metabolic processes, immune responses, and disease-associated phenotypes. Seminal studies were also included to provide historical background and conceptual context. ClinicalTrials.gov records were additionally consulted to identify the development stage of STC-15, with status checked on 15 October 2026. As this article represents a narrative review, the literature search was performed to support thematic synthesis and conceptual integration rather than a formal systematic review or meta-analysis.

## 2. m^6^A and Metabolic Programming of Innate Immune Cells

### 2.1. Macrophages: m^6^A as a Metabolic Switch Controlling Inflammatory and Reparative States

Macrophages adapt their functions and metabolism to environmental signals. In commonly used experimental models, inflammatory M1-like activation is often accompanied by increased glycolysis, whereas IL-4-associated M2-like activation can involve oxidative metabolism and fatty acid utilization [20,25,26]. These patterns provide a useful starting point for discussing how m^6^A-dependent RNA regulation influences macrophage responses.

The M1/M2 distinction is a simplified description of stimulation-dependent features, rather than a complete classification of tissue macrophages. Single-cell analysis has identified lipid-associated macrophage states in adipose tissue, including TREM2-dependent programs linked to lipid handling [27]. Tissue-resident, inflammatory, reparative, fibrotic, and tumor-associated states can overlap and need not follow a fixed glycolysis-versus-FAO division. Accordingly, the M1-like and M2-like labels below refer to the phenotypes measured in each study; they should be interpreted together with tissue context, stimulation conditions, and the metabolic measurements available.

#### 2.1.1. m^6^A-Driven Glycolytic Reprogramming in Inflammatory Macrophages

During inflammatory activation, macrophages undergo a metabolic transition characterized by enhanced glycolysis and accumulation of glycolytic intermediates. Studies in specific disease models link m^6^A-dependent RNA regulation to these glycolytic responses. In a sepsis model, hypoxia-inducible factor-1α (HIF-1α) increased METTL3 expression and m^6^A enrichment on phosphofructokinase M (PFKM) RNA, accompanied by increased PFKM protein expression. METTL3 silencing attenuated this response, and functional experiments linked PFKM to macrophage glycolysis and inflammatory cytokine production [28]. Similarly, METTL3-mediated m^6^A modification stabilizes hepatoma-derived growth factor (HDGF) mRNA, and HDGF promotes M1-like macrophage polarization through metabolic reprogramming, thereby accelerating atherosclerotic progression [29].

m^6^A regulation also affects additional glycolytic rate-limiting enzymes. In necrotizing enterocolitis, reduced m^6^A modification of *PFKFB3* mRNA increases its expression, promoting glycolysis and driving macrophages toward an inflammatory phenotype [30]. These studies support a role for m^6^A-dependent regulation of glycolytic transcripts in inflammatory macrophage responses in the models examined. Conversely, demethylases can regulate inflammatory activation by modifying the stability of metabolic regulators. In diabetic nephropathy, reduced FTO expression increases m^6^A modification of *Npas2* mRNA, resulting in activation of the HIF-1α signaling pathway and enhanced macrophage glycolysis and inflammation [31]. In obesity-associated inflammation, free fatty acids and lipopolysaccharide (LPS) synergistically increase FTO expression, leading to accelerated degradation of *CSF1* mRNA through m^6^A demethylation and promoting M1 polarization and insulin resistance [32]. Together, these models link changes in RNA regulation to macrophage metabolism and inflammatory responses, with the direction of the effect depending on the stimulus and target transcript.

#### 2.1.2. m^6^A-Mediated Lipid Metabolic Remodeling During Alternative Macrophage Activation

During alternative macrophage activation, oxidative metabolism and lipid utilization can support reparative functions, although the relationship varies by tissue and stimulus. The cellular origin of an m^6^A pathway is particularly important in tumors. In triple-negative breast cancer cells, D-2HG inhibits FTO and increases m^6^A-dependent, YTHDF1-mediated translation of ANGPTL4. Secreted ANGPTL4 then promotes macrophage M2-like polarization through paracrine signaling [33]. This is a tumor cell mechanism that influences macrophages. By comparison, in a myocardial infarction model, alpha-ketoglutarate (α-KG) supplementation supports FTO-mediated regulation of STAT3 in macrophages, promoting anti-inflammatory activation and cardiac repair [34]. These findings illustrate how the same enzyme can connect distinct metabolic inputs to different cellular targets.

Additional studies support m^6^A-dependent regulation within macrophages. In diabetic vasculopathy models, FTO stabilizes target transcripts through an m^6^A-YTHDF2-dependent mechanism and supports PI3K/AKT signaling and macrophage homeostasis [35]. WTAP-dependent regulation in adipose tissue macrophages provides another example of context-dependent metabolic control and is discussed in Section 4.2 [36]. ALKBH5-mediated regulation of CPT1A promotes fatty acid metabolism and M2-like polarization in colorectal cancer-associated macrophages [37]. In pulmonary fibrosis models, IGF2BP1 stabilizes *THBS1* mRNA and promotes macrophage glycolytic activity and a fibrotic phenotype [38]. These results cannot be reduced to a common inflammatory effect of m^6^A deposition or removal. FTO-dependent Npas2 regulation limits inflammatory glycolysis in diabetic nephropathy [31], whereas the FTO-CSF1 pathway promotes inflammatory polarization during fatty acid and LPS exposure [32]. The differences are consistent with changes in the stimulus and target transcript; disease stage is an additional variable that requires direct comparison, rather than an established explanation for every opposing result.

#### 2.1.3. Disease-Associated Macrophage Metabolic Remodeling by m^6^A Modification

The interaction between m^6^A regulation and macrophage metabolism has important implications in cancer, metabolic disorders, and cardiovascular disease. In cancer, tumor-associated macrophages (TAMs) frequently acquire immunosuppressive metabolic programs characterized by enhanced FAO and mitochondrial metabolism. In hepatocellular carcinoma, tumor-derived exosomal SLC16A1-AS1 enhances *SLC16A1* mRNA stability in macrophages, increasing lactate uptake and promoting M2 polarization through c-Raf/ERK signaling. Meanwhile, IL-6 produced by M2 macrophages enhances METTL3 activation in tumor cells, establishing a positive feedback loop that reinforces tumor progression [39]. In colorectal cancer models, tumor-derived lactate increases histone H3K18 lactylation and METTL14 expression in macrophages. METTL14 and the reader YTHDC1 support increased VSIG4 mRNA stability. VSIG4 promotes FAO and M2-like polarization through JAK2/STAT3-associated signaling [40]. In glioma, loss of METTL3 enhances macrophage lipid metabolism through the ISG15-FASN pathway and promotes tumor progression [41].

**Figure 2:**
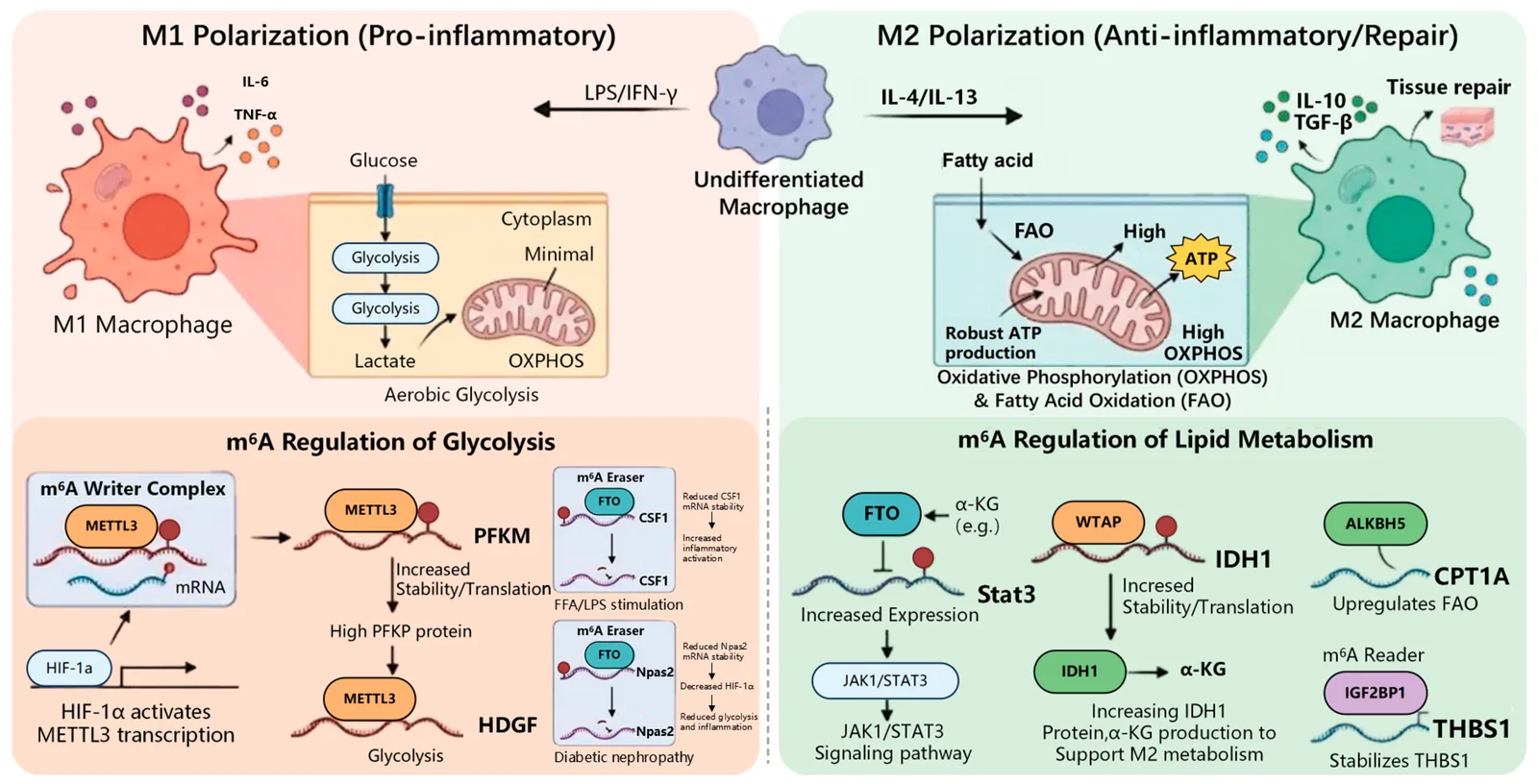
m^6^A modification regulates macrophage metabolic reprogramming and polarization.

Outside tumors, obesity-associated FTO upregulation impairs pri-miR-192 processing and increases macrophage activation in experimental acute lung injury [42]. In atherosclerosis models, 3-hydroxystearic acid has been reported to modulate the ALKBH5/PAX8/ABCA1 pathway, increasing cholesterol efflux and reducing foam cell formation [43]. The broader literature on FTO and lipid metabolism provides context for these findings but cannot by itself establish a macrophage-specific protective mechanism [44]. Together with the WTAP-IDH1-α-KG and FTO pathways discussed above, these studies support context-dependent regulation of inflammatory and reparative macrophage features (Figure 2).

Endometriosis provides a further example of stromal–immune communication. Gou et al. combined clinical associations with cell and mouse experiments to show that lactate from ectopic endometriotic stromal cells promotes M2-like macrophage polarization through an METTL3/TRIB1/ERK/STAT3-associated pathway [45]. A separate METTL14/YTHDF2/KLF4 study used tissue analyses, stromal cell/macrophage coculture, and rescue experiments to link RNA regulation to macrophage polarization [46]. These findings concern macrophage responses within an inflammatory lesion; their applicability beyond the tested lesion and coculture models remains to be established.

### 2.2. Dendritic Cells: m^6^A Regulation of Metabolic Fitness and Antigen-Presenting Function

Dendritic cells (DCs) link innate and adaptive immunity through signaling, antigen presentation, and migration. PAR1-S1P3 signaling illustrates their role in coupling coagulation to inflammation [47], whereas YTHDF1-dependent RNA regulation affects antigen presentation [48]. Direct links between RNA regulation and DC metabolism have been examined in the lnc-Dpf3 migration and METTL3 corneal transplant studies discussed below [49,50].

During DC migration, metabolic reprogramming toward enhanced glycolysis provides the energy and biosynthetic resources required for cytoskeletal remodeling and lymph node trafficking. A representative example is the regulation of lnc-Dpf3 by m^6^A remodeling. Following CCR7 stimulation, demethylation of lnc-Dpf3 increases its RNA stability, establishing a negative feedback mechanism that limits DC metabolic activation. Mechanistically, stabilized lnc-Dpf3 interacts with HIF-1α and suppresses transcription of glycolytic genes such as LDHA, thereby reducing glycolytic metabolism and impairing DC migration [49]. This finding demonstrates that m^6^A regulation can indirectly shape immune cell metabolism through non-coding RNA-mediated transcriptional control. The m^6^A methyltransferase METTL3 also contributes to DC metabolic programming. In a corneal allograft rejection model, METTL3 expression is increased following immune activation and promotes glycolytic metabolism and inflammatory cytokine production through activation of the mTOR pathway. Conditional deletion of *Mettl3* in DCs or pharmacological inhibition with STM2457 reduces glycolytic activity, suppresses inflammatory responses, and delays graft rejection [50]. These findings identify METTL3 as a metabolic regulator linking epitranscriptomic modification with DC-mediated immune activation.

The lnc-Dpf3 and corneal transplant studies combine functional perturbations with measurements of DC glycolysis [49,50]. Disease-associated expression and genetic analyses provide complementary hypotheses, but do not directly establish the proposed m^6^A-dependent metabolic mechanisms in DCs [51,52,53,54]. Their designs and evidence boundaries are summarized in Table 1. Cell-specific perturbation, target validation, and metabolic measurements are needed to connect these associations to defined DC functions.

### 2.3. NK Cells: m^6^A-Mediated Metabolic Adaptation and Cytotoxic Function

Natural killer (NK) cells require metabolic adaptation to sustain cytokine responses and cytotoxicity [55]. Reviews of RNA modification in immune regulation describe roles for m^6^A writers and readers in NK cell activation and cytokine responsiveness [21]. These observations provide a rationale for studying NK immunometabolism, although changes in immune signaling or cytotoxicity do not by themselves demonstrate altered glycolytic or mitochondrial flux.

Direct functional evidence is available for FTO. Kim et al. found that FTO stabilizes SOCS family transcripts, including SOCS3, and restrains IL-2/IL-15-driven JAK/STAT signaling [56]. FTO-deficient human NK cells showed enhanced cytotoxicity in vitro, and *Fto*-deficient mouse NK cells reduced melanoma metastasis in vivo. These data support an NK cell-intrinsic signaling and effector mechanism, while its metabolic consequences require direct flux measurements. Lactate-dependent regulation of m^6^A machinery has been reviewed across several disease settings [57], but an NK cell-specific lactate–H3K18la-METTL3 pathway requires direct validation before it can be used to explain NK dysfunction.

Current NK cell evidence is strongest for the coupling of m^6^A regulation to cytokine signaling and cytotoxic activity. Resolving how these pathways affect nutrient use, mitochondrial respiration, and sustained antitumor function will require metabolic measurements alongside cell-specific perturbation.

### 2.4. Pharmacological Targeting of m^6^A Modifiers in Innate Immune Cell Metabolic Reprogramming

Pharmacological studies offer opportunities to modify m^6^A-associated immune responses, but their implications depend on the type of intervention and the model used. Enzyme inhibitors, delivery systems, complex herbal formulas, and vitamin-based interventions differ in target selectivity and supporting evidence. Cellular target engagement, a functional response in an animal model, and an association with clinical improvement address separate questions and should be evaluated separately.

STM2457 has been tested in several preclinical settings. In a pulmonary hypertension mouse model, STM2457 treatment and myeloid *Mettl3* deletion reduced inflammatory responses, pulmonary vascular remodeling, and right heart remodeling [58]. The evidence summarized here concerns inflammatory and hemodynamic endpoints. In alveolar echinococcosis models, STM2457 reduced ACSL4-associated ferroptotic changes, macrophage M2-like polarization, and fibrotic remodeling [59]. In colorectal cancer, an STM2457-loaded hydrogel altered tumor cell chemokine output and improved CAR-NK recruitment and tumor control in preclinical experiments [60]. This delivery approach acts through the tumor environment and should not be interpreted as evidence of a CAR-NK cell-intrinsic metabolic mechanism or established clinical safety.

Xinfeng Capsule (XFC) illustrates why clinical and mechanistic evidence need to be separated. Sun et al. reported a retrospective cohort of 2367 patients with rheumatoid arthritis, with propensity score-matched comparisons of inflammatory indices and liver/kidney safety measures [61]. These observations were complemented by in vitro studies of the ALKBH5-m^6^A-LINC00968 pathway, neutrophil activation, oxidative stress, and NETosis, including perturbation and rescue experiments. The clinical analysis remains observational; the metabolic and RNA regulatory mechanisms were investigated in vitro. Proposed binding of formula constituents to ALKBH5 was supported by molecular docking and requires direct experimental target engagement validation.

Together, these studies provide preclinical rationale for targeting m^6^A regulators and preliminary clinical associations for a broader intervention. XFC should therefore be described as an m^6^A-associated formula, rather than a validated selective ALKBH5 inhibitor. Translation will require prospective clinical assessment, direct target engagement studies, and delivery strategies that account for the distinct effects of the same regulator in different cell populations [58,59,60,61].

## 3. m^6^A and Metabolic Adaptation of Adaptive Immune Cells

### 3.1. T Lymphocytes

T cell activation and differentiation are accompanied by metabolic adaptation [62,63]. Studies of m^6^A regulation identify effects on transcriptional regulators, signaling, and metabolic genes [64], although the contribution of a directly measured metabolic change varies among models.

#### 3.1.1. CD8^+^ T Cells: m^6^A Regulation of Activation, Effector Function, Memory and Exhaustion

CD8^+^ T cell activation, effector differentiation, and memory formation require coordinated metabolic adaptation [65]. T cell-intrinsic m^6^A regulation contributes to differentiation and maintenance, as shown by *Mettl3* perturbation studies [66,67]. In a separate study, T cell-specific *Fto* deletion increased m^6^A modification and IGF2BP3-dependent stabilization of *Fas* mRNA, promoting apoptosis of activated CD8^+^ T cells [68]. Fas m^6^A site mutation and IGF2BP3 knockdown provided additional support for this RNA-dependent mechanism. This study establishes a survival pathway; it does not by itself demonstrate a change in T cell metabolic flux.

Tumor cell m^6^A regulation can affect CD8^+^ T cells indirectly. In hypoxic breast cancer cells, HIF-1α induces FTO, which reduces m^6^A modification of *PDK1* mRNA and limits YTHDF3-associated transcript degradation. The resulting PDK1/AKT/STAT3 signaling increases PD-L1 expression and suppresses T cell cytotoxicity [69]. In another tumor model, loss of tumor cell YTHDF2 reduced tumor glycolytic activity and enhanced mitochondrial respiration in infiltrating CD8^+^ T cells [70]. These findings locate the initial m^6^A perturbation in tumor cells and the downstream immune effect in T cells.

Metabolite exchange and extracellular vesicles provide additional routes of intercellular regulation. In gastric cancer cells, m^6^A modification stabilizes 3′tRF-AlaAGC, promoting PTBP1-associated glycolysis and lactate production. Treg cells take up lactate through MCT1, with NFAT1 nuclear translocation and increased PD-1 expression contributing to an immunosuppressive environment [71]. In hepatocellular carcinoma, circPETH is transferred from tumor-associated macrophages to tumor cells through extracellular vesicles. Its m^6^A-dependent translation produces circPETH-147aa, which promotes tumor glycolysis and HuR-dependent stabilization of *SLC43A2* mRNA, increasing competition for methionine and leucine and impairing CD8^+^ T cell function [72]. The demonstrated RNA and metabolic events in these examples occur mainly in tumor cells, with downstream effects on neighboring lymphocytes.

A separate analysis of renal clear cell carcinoma linked FTO and METTL3 expression and DNA methylation to patient survival and chemokine expression patterns [73]. These are prognostic and molecular associations. They do not establish a CD8^+^ T cell-intrinsic mechanism, nor do they validate either regulator as a predictor of response to immunotherapy.

#### 3.1.2. CD4^+^ T Cells: m^6^A Regulation of Subset Differentiation and Metabolic Programs

CD4^+^ T cell subsets, including Th1, Th17, and regulatory T cells, adopt distinct metabolic programs during differentiation and activation [62,63,74]. Experimental studies link m^6^A regulators to their lineage programs, including METTL14-dependent maintenance of FOXP3 in induced Treg cells and METTL3-dependent regulation of pathogenic Th17 cells [75,76]. The lupus nephritis study reported both an m^6^A-dependent RORA branch and an m^6^A-independent Smad2/STAT3-RORC branch, illustrating why regulator effects need not all arise from RNA methylation [76]. T cell-specific *Fto* deletion impairs Th1 differentiation and antimicrobial immunity in a separate study [77]. These findings support cell-intrinsic regulation of differentiation, while the contribution of metabolic flux must be judged from the measurements made in each model.

In asthma models, the METTL3/miR-192-5p/SCD1 pathway connects RNA regulation to lipid metabolism and T cell differentiation. Inhibition of miR-192-5p increased SCD1-associated lipid metabolic signals and Th2/Th17 differentiation, whereas SCD1 inhibition reversed these effects; METTL3 overexpression reduced lung inflammation and Th2/Th17 responses [78]. In Graves’ ophthalmopathy, a small patient sample study combined RNA-seq and MeRIP-seq with CD4^+^ T cell perturbation experiments. WTAP silencing reduced THBS1 m^6^A enrichment and expression, and THBS1 silencing reduced glucose uptake and lactate measurements while affecting the Th17/Treg balance [79]. These data support a cellular mechanism alongside clinical observations, but do not establish treatment efficacy in patients.

An additional study linked IL-37 signaling to CD4^+^ T cell glycolysis in colitis models [19]. IL-37 acted through SIGIRR and the IRAK4/JNK/NF-κB pathway to reduce METTL14 expression and m^6^A modification of SLC2A1 RNA. RNA stability assays and analysis of the SLC2A1 A2445 site supported a transcript-specific mechanism, while extracellular acidification and glucose uptake measurements demonstrated reduced glycolytic activity. These changes were associated with reduced Th1/Th17 differentiation and increased Th2 differentiation. Perturbation and adoptive transfer experiments support this pathway in experimental models, but do not establish treatment efficacy in patients.

m^6^A regulation can also influence CD4^+^ T cell differentiation through immune cell crosstalk. In colitis models, myeloid Mettl3 deletion and macrophage experiments linked reduced YTHDF3-mediated decay of Pgp mRNA to increased PGP expression, pentose phosphate pathway (PPP)-associated metabolism, and mitochondrial respiration. Glycolysis was not detectably altered in the reported Seahorse assays. Macrophage coculture and in vivo experiments connected these changes to reduced Th1 differentiation and intestinal inflammation [80]. Furthermore, glycolysis-driven m^6^A remodeling regulates Tfh cell development. VHL deficiency enhances glycolysis through HIF-1α stabilization, while increased GAPDH-dependent m^6^A modification reduces ICOS expression and suppresses early Tfh differentiation [81]. This study links glycolysis-associated m^6^A regulation to early Tfh differentiation in the VHL-deficient model.

**Figure 3:**
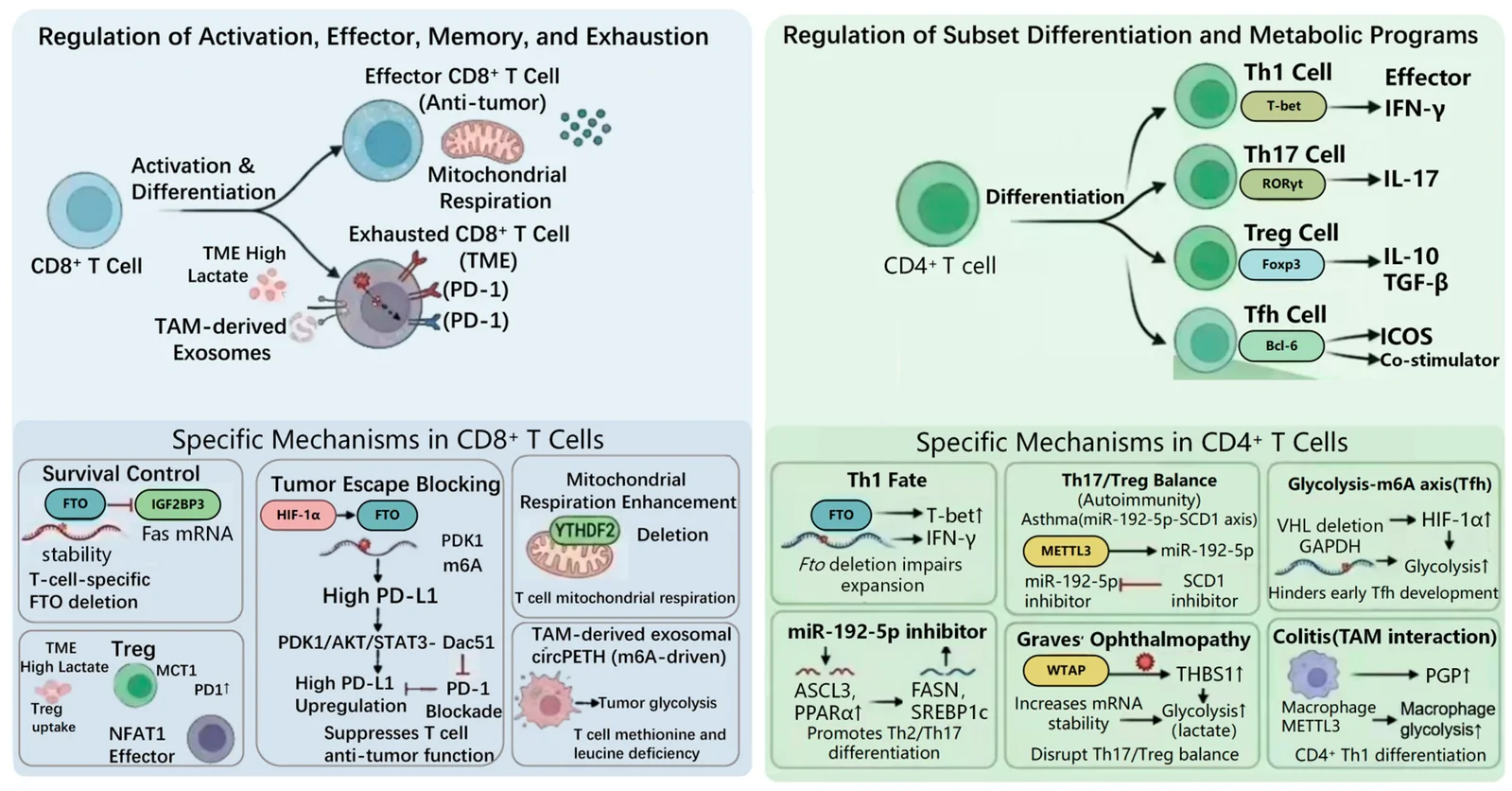
m^6^A-associated regulation of T cell responses. The mechanisms summarized include T cell-intrinsic pathways and signals originating in tumor cells or macrophages; differentiation, survival, and immune responses should be distinguished from directly measured metabolic changes. *Connecting arrows indicate the direction of the depicted pathways or regulatory effects; blunt-ended lines indicate inhibition. Upward and downward arrows next to labels indicate increases and decreases, respectively.*

Collectively, these studies link m^6^A regulation to CD4^+^ T cell differentiation through cell-intrinsic pathways and macrophage–T cell communication; direct metabolic endpoints are established in selected models. However, the biological meaning of each m^6^A regulator remains context-dependent: the same enzyme may reinforce effector differentiation, immune tolerance, or inflammatory pathology depending on the T cell subset, antigenic stimulus, metabolic stress, and target transcript involved (Figure 3).

### 3.2. B Lymphocytes

In normal B cells, m^6^A regulation has been linked to development, pre-B cell expansion, and antibody responses [22,82]. These processes require metabolic support, but evidence for developmental or class-switching phenotypes should be distinguished from direct measurements of metabolic reprogramming. The literature summarized in these reviews does not establish a single m^6^A-dependent metabolic pathway that accounts for all stages of normal B cell differentiation.

In malignant B cells, several studies provide more direct metabolic evidence. Chen et al. showed that YTHDF2 has distinct reader activities: it stabilizes m5C-modified transcripts involved in ATP synthesis through PABPC1, while its m^6^A reader activity promotes the decay of transcripts involved in antigen presentation and immune recognition [83]. Thus, the ATP synthesis and immune evasion branches should not both be attributed to m^6^A. In chronic lymphocytic leukemia, m^6^A-modified circTET2 interacts with HNRNPC to regulate CPT1A mRNA stability, fatty acid oxidation, and mTORC1-associated proliferation [84]. In B cell acute lymphoblastic leukemia, FTO perturbation affects ribosomal protein transcripts, nucleolar stress, and mitochondrial function; FTO inhibition also enhanced the response to doxorubicin in preclinical models [85]. These effects were examined in malignant B cells and cannot be assumed to apply to normal B cell metabolism.

Other B cell malignancy studies establish oncogenic signaling without directly establishing metabolic flux. In diffuse large B cell lymphoma, FTO-dependent stabilization of FLOT2 activates PI3K/Akt/mTOR signaling, while KIAA1429/YTHDF2-dependent destabilization of CHST11 alters Hippo-YAP signaling [86,87]. Perturbation and rescue experiments support these RNA-to-signaling pathways, but describing them as demonstrated metabolic reprogramming would exceed the evidence presented.

In autoimmune B cells, the TLR7-FTO-ATP6V1G1 pathway provides a more direct link to mitochondrial metabolism. Zeng et al. combined observations in patients with systemic lupus erythematosus with human and mouse B cell experiments and lupus models [88]. FTO supports ATP6V1G1-dependent lysosomal function, mitochondrial quality control, and OXPHOS during age-associated B cell differentiation. The patient association and the experimental B cell-intrinsic mechanism are complementary evidence, while clinical benefit from targeting this pathway remains to be established.

### 3.3. Pharmacological Targeting of m^6^A Regulators and Clinical Development

Pharmacological studies in adaptive immune contexts include inhibition of alloreactive T cells, targeting of malignant lymphocyte metabolism, and tumor cell interventions that may influence immunity. Their disease models and cellular targets should be kept distinct when evaluating therapeutic relevance.

In an allogeneic response study, STM2457 reduced m^6^A levels, proliferation, and Th1 differentiation in stimulated CD4^+^ T cells [89]. TEa cells are T cell receptor transgenic CD4^+^ T cells specific for the I-Eα52-68 peptide presented by I-A^b^. In the reported experiments, STM2457 increased apoptosis and reduced proliferation-associated Ki-67 and the transcription factors c-Myc and T-bet. These results support suppression of alloreactive T cell responses; they should not be described as evidence for enhanced antitumor immunity or for a directly measured metabolic switch.

In T- cell acute lymphoblastic leukemia, FTO stabilizes *ELK3* mRNA and promotes expression of glycolytic genes. Dac51 suppressed this pathway and showed antileukemic activity in cell and animal models [90]. This is a malignant T cell metabolic mechanism, rather than evidence about normal effector T cell metabolism. SKLB-Y13 provides a separate example of reader inhibition: biochemical, target engagement, cellular, and animal studies support selective inhibition of YTHDF1 and disruption of *PRPF6* mRNA translation in breast cancer models [91]. Its activity was demonstrated in tumor cells and should not be presented as validated T cell metabolic therapy.

METTL3 inhibition has also entered clinical investigation. The first-in-human phase 1 study of oral STC-15 in advanced malignancies (NCT05584111) is registered as completed [92]. A subsequent phase 1b/2 study (NCT06975293) is registered as recruiting and evaluates STC-15 in combination with toripalimab in selected advanced cancers, with additional STC-15 monotherapy cohorts in selected sarcomas [93]. These registry statuses were checked on 15 October 2026. They establish the stage of clinical development, but do not establish clinical benefit, long-term safety, or an immune cell-intrinsic metabolic mechanism. Neither registry record displayed posted results at the time of this check.

The experimental studies above support distinct preclinical uses of m^6^A-targeting compounds, while STC-15 illustrates early clinical development in oncology. These findings do not establish a common therapeutic effect across adaptive immune diseases. Clinical benefit, cellular selectivity, and long-term safety for the immunometabolic applications discussed remain to be established.

## 4. m^6^A Regulation in Tissue-Resident and Specialized Immune Cells

m^6^A regulation has also been investigated in tissue-resident immune populations and specialized tissue environments [23,24]. The studies below address inflammatory signaling, cellular stress, and selected metabolic pathways; not all demonstrate altered metabolic flux.

### 4.1. Microglia: m^6^A Regulation of Neuroinflammation and Neurodegeneration

Microglia are the resident immune cells of the central nervous system and play essential roles in neuronal maintenance, inflammatory responses, and tissue repair. Their functional polarization is closely associated with metabolic remodeling, including changes in mitochondrial activity, oxidative metabolism, lipid utilization, and inflammatory signaling. Studies in specific injury and inflammatory models link m^6^A-dependent RNA regulation to microglial signaling, activation, and cell survival. In cerebral ischemia–reperfusion injury, microglial m^6^A modification levels are increased, whereas expression of the demethylase FTO is reduced. Restoration of FTO activity or inhibition of excessive m^6^A modification alleviates microglia-mediated inflammatory damage. Mechanistically, FTO promotes degradation of *cGAS* mRNA through m^6^A regulation, thereby suppressing activation of the STING/NF-κB inflammatory pathway [94]. Similarly, FTO-mediated regulation of *GIV* mRNA activates Wnt/β-catenin signaling and inhibits excessive microglial activation and M1 polarization [95]. In addition, HOXA5-induced FTO expression decreases m^6^A modification of *TMEM59* mRNA, preventing YTHDF2-dependent transcript degradation and reducing microglial pyroptosis after stroke [96]. Intercellular communication can also influence microglial RNA regulation and inflammatory responses. Neural stem cell-derived exosomes can deliver FTO into microglia, reducing m^6^A modification of *NRF2* mRNA and promoting M2-like polarization, thereby protecting neurons from inflammatory injury [97]. These findings support context-dependent effects on microglial signaling and inflammatory injury, rather than a shared, directly measured metabolic mechanism.

Metabolic stress also affects microglial m^6^A regulation. In high-glucose models, reduced ALKBH5 expression increases m^6^A enrichment and degradation of *A20* mRNA, favoring inflammatory microglial activation; increasing ALKBH5 protects A20 transcripts from degradation [98]. In a distinct endothelial cell mechanism, lactate-driven histone lactylation increases FTO expression and alters CDK2 RNA stability, with downstream endothelial–microglial interactions contributing to retinal injury [99]. Arsenic exposure models connect reduced FTO and increased ATG9A m^6^A modification to ferritinophagy and microglial activation [100]. In cell and mouse models of LPS-induced neuroinflammation, vitamin D acts through VDR-dependent transcriptional induction of FTO and an FTO/Mxd1/PTEN/AKT/PGC-1α pathway [101]. This is evidence for an indirect, context-specific modulation of m^6^A regulation, not for vitamin D as a selective FTO-targeting drug. Finally, YTHDF2 knockdown stabilizes PARP14 and attenuates oxygen glucose deprivation-induced microglial inflammation in a cellular model [102]. The experimental settings and cellular origins differ across these studies and limit direct extrapolation to clinical neuroinflammatory disease.

These microglial studies connect RNA regulation to inflammatory signaling, iron handling, and responses to metabolic stress. Some endpoints concern immune activation or cell injury rather than measured energy flux; the distinction matters when interpreting them as evidence for immunometabolic reprogramming.

### 4.2. Adipose Tissue-Associated Immune Cells

Reduced WTAP expression in adipose tissue macrophages was associated with adverse metabolic characteristics in people with obesity [36]. In experimental models, LysM-Cre-mediated myeloid Wtap deletion aggravated high-fat-diet-induced metabolic dysfunction, while macrophage experiments supported WTAP-dependent stabilization and translation of IDH1 mRNA, increased α-KG production, and alternative activation. These data support a macrophage mechanism within the studied models, although the conditional deletion is not restricted exclusively to macrophages and the human observations do not establish clinical benefit from changing WTAP.

### 4.3. Kupffer Cells in Metabolic Liver Disease

In activated Kupffer cells, NF-κB-dependent induction of METTL3/METTL14 promotes m^6^A-dependent TGF-β1 translation. Experiments in Kupffer cells and myeloid *Mettl14*-deficient mice support a role in inflammatory and fibrotic liver responses [103]. A separate FTO/IL-17RA study combined human liver tissue observations with hepatic cell and mouse experiments [104]. It supports a mechanism in chronic liver inflammation, but does not establish that the initiating FTO perturbation is Kupffer cell-specific. The latter study should therefore be interpreted as evidence from the wider hepatic microenvironment, rather than as a second demonstrated Kupffer cell-intrinsic pathway.

Table 2 summarizes representative pathways across immune populations, distinguishing cellular origin, metabolic or immune endpoints, and the experimental evidence supporting each connection.

## 5. Metabolite–m^6^A Interactions in Immune Regulation

The studies above suggest a reciprocal relationship: metabolic inputs can alter m^6^A regulation, while RNA regulation can change metabolic enzymes, transporters, and immune responses. The strength of evidence differs for the two directions and for different cell populations. The following synthesis focuses on these relationships and their implications for interpreting disease models.

### 5.1. Metabolic Regulation of m^6^A Machinery

Metabolites influence m^6^A pathways through more than one route. α-KG is a cosubstrate for FTO and ALKBH5, whereas D-2HG can inhibit FTO in a defined tumor context [2,33,34]. Lactate-associated histone lactylation can alter transcription of m^6^A regulators [40,57,99]. A change in enzyme expression should be distinguished from a direct change in catalytic activity, and neither alone identifies the RNA target responsible for an immune phenotype.

The downstream response depends on the responding cell and target transcript. A metabolite may act on an immune cell directly or change a neighboring tumor or stromal cell that subsequently releases an immune-modulating signal. The tumor cell ANGPTL4 pathway and the macrophage WTAP-IDH1 pathway illustrate these different cellular arrangements [33,36]. This distinction is essential when attributing a metabolic response to immune cell-intrinsic m^6^A regulation.

Reciprocal regulation does not necessarily constitute a closed feedback loop in every model. Demonstrating such a loop requires perturbation of both the metabolic input and the RNA regulatory output in the relevant cells. Pathways that have been established separately can guide hypotheses about feedback, but should not be joined into a fully validated circuit without direct evidence.

### 5.2. Global Metabolic States Reshape the m^6^A Landscape

Hypoxia, nutrient limitation, and metabolite accumulation can affect the expression or activity of RNA regulators. HIF-1α-dependent mechanisms have been described in macrophage inflammatory models and in hypoxic breast cancer cells [28,69]. Their immune consequences differ because the initiating cell and downstream transcript differ. More general proposals concerning oxidative stress or nutrient deprivation require target-specific testing rather than inference from the metabolic state alone.

These global metabolic inputs should be interpreted with caution because many studies do not yet distinguish direct m^6^A-dependent effects from broader stress responses. Future work should combine quantitative m^6^A mapping, target site validation, metabolic flux analysis, and rescue experiments to determine whether a phenotype is driven by a specific m^6^A-marked transcript or by m^6^A-independent functions of the same regulator.

### 5.3. Integrated Disease Models

#### 5.3.1. Metabolic Stress and Macrophage State

Obesity-related models link nutrient excess to several RNA regulatory pathways, but the outcomes depend on the experimental exposure and macrophage population [32,36,42]. These studies favor a state-based interpretation over a universal inflammatory function for FTO or WTAP. Direct comparisons across tissues and time points will be needed to determine whether distinct transcript targets account for the observed differences.

#### 5.3.2. Metabolite Exchange in Tumor Immunity

Tumor-associated metabolic competition can connect m^6^A regulation in one cell to immune dysfunction in another [40,70,71,72]. The initiating event may involve tumor glycolysis, macrophage signaling, or extracellular vesicle transfer, with different consequences for effector and regulatory lymphocytes. Therapeutic interpretation therefore depends on identifying the cell in which a regulator must be perturbed and measuring both tumor and immune responses. The cited studies support individual components of this network; they do not establish one shared lactate-driven m^6^A mechanism in all tumor-infiltrating immune cells.

These comparisons suggest that cellular origin, target transcript, and metabolic context jointly determine the outcome of an m^6^A intervention. They also identify practical gaps: distinguishing direct from indirect effects, testing whether proposed feedback operates within the same model, and establishing the consequences of cell-selective delivery.

Representative preclinical interventions, an observational clinical study, and registered STC-15 trials relevant to m^6^A-associated immune regulation are summarized in Table 3. The table distinguishes the cellular target, observed or planned endpoint, and evidence supporting each intervention.

### 5.4. Technical Limitations and Unresolved Questions

Antibody-based MeRIP-seq detects enriched RNA regions rather than the modification status of every individual adenosine. Antibody specificity, RNA abundance, sequencing depth, and analysis choices can affect reproducibility and the interpretation of differential peaks [105]. A change in an MeRIP signal should therefore be assessed alongside input RNA and orthogonal validation. Regulator knockdown or knockout alone does not establish that a phenotype is caused by a particular m^6^A site.

Substrate and reader specificity introduce further uncertainty. FTO can act on cap-adjacent m^6^Am as well as internal m^6^A, so FTO-dependent phenotypes are not automatically evidence for internal m^6^A regulation [2,106]. IGF2BP proteins have broader RNA-binding functions, and m^6^A dependence needs to be demonstrated for the specific transcript and phenotype under study [3]. The dual m5C/m^6^A activities reported for YTHDF2 in B cell malignancies provide another reason to separate regulator-level effects from modification-specific mechanisms [83]. Studies proposing a *shared* mRNA decay function for YTHDF proteins and context-dependent compensation also caution against assuming either complete redundancy or a fixed division of labor for YTHDF1/2/3 in every immune population [107,108].

Mechanistic confidence is increased by convergent evidence from target site mutation, catalytic mutant rescue, reader dependency testing, direct RNA-binding assays, and measurements of the proposed metabolic output. Metabolic flux measurements are particularly important because immune activation, proliferation, or pathway enrichment can change without establishing the claimed metabolic mechanism. These criteria should be applied to individual claims rather than used to rank an entire study as simply mechanistic or correlative.

## 6. Conclusions and Future Perspectives

m^6^A-dependent RNA regulation contributes to immune function through pathways involving metabolic enzymes, nutrient transport, cytokine signaling, and cell survival. The strongest evidence comes from models that connect a defined RNA target to a measured metabolic or immune phenotype using perturbation and rescue experiments.

The effects of individual regulators vary across immune lineages and tissue environments. Macrophage studies show that the direction of inflammatory change depends on the stimulus and target transcript; lymphocyte studies distinguish survival and differentiation mechanisms from direct metabolic effects; and tumor or stromal mechanisms can alter immune function through intercellular communication. These differences argue for evaluating each pathway in its cellular setting.

Evidence also remains uneven. Expression correlations, enrichment analyses, and genetic inference generate testable hypotheses, whereas target site experiments and cell-specific genetic models offer stronger support for defined mechanisms. Even mechanistic findings in one disease model do not establish general clinical efficacy or safety.

Future studies should combine quantitative RNA modification mapping with metabolic flux analysis and cell-specific perturbation. Pharmacological development will require direct target engagement measurements, selective delivery, and prospective clinical evaluation. These steps will help identify when altering an m^6^A regulator can improve immune function without compromising protective responses in other cells.

## Figures and Tables

**Table 1:** Designs and evidence boundaries of disease-associated studies relevant to DCs.

Study	Design and Validation	Supported Inference	Mechanistic Gap
Ligamentum flavum ossification [51]	Transcriptomics, inferred immune infiltration, RT-qPCR and Western blots	Differential expression and inferred immune cell abundance	No direct test of m^6^A-dependent DC metabolism
Dilated cardiomyopathy [52]	Public expression datasets, enrichment and immune cell deconvolution	Associations among metabolic genes, m^6^A regulators and inferred infiltration	No DC-specific perturbation or metabolic measurement
Lung adenocarcinoma [53]	Molecular datasets, pathway/infiltration analysis, RT-qPCR and Western blots	STARD12/14 expression and molecular associations	No experimentally validated DC-specific m^6^A metabolism pathway
Dental caries [54]	Genetic association, m^6^A-QTL, single-cell analysis and two-sample Mendelian randomization	Genetic causal inference conditional on instrumental variable assumptions	No experimental validation of m^6^A-ABHD12-OXPHOS in DCs

**Table 2:** Representative m^6^A-associated metabolic and immune pathways across cellular contexts.

Immune Cell or Context	m^6^A Regulator	Representative Target	Metabolic or Immune Endpoint	Cellular Origin and Interpretation	References and Evidence
Macrophages	METTL3; m^6^A remodeling	PFKM, HDGF, PFKFB3	Glycolysis and inflammatory activation	Macrophage mechanisms; the regulator of PFKFB3 m^6^A loss is not assumed to be METTL3	Functional and metabolic studies [28,29,30]
Macrophages and tumor cells	FTO	Npas2, CSF1, ANGPTL4, STAT3	Glycolysis, inflammation, polarization, cardiac repair	Npas2/CSF1/STAT3: macrophage pathways; ANGPTL4: tumor cell signal to macrophages	Perturbation studies; distinct cellular origins [31,32,33,34,35]
Adipose tissue macrophages	WTAP	IDH1	α-KG production and alternative activation	Human association; myeloid conditional deletion and macrophage target studies	Human association and experimental mechanism [36]
Tumor-associated macrophages	METTL14; YTHDC1	VSIG4	Fatty acid oxidation and polarization	Tumor-derived lactate acts on macrophage RNA regulation	Preclinical mechanism [40]
Dendritic cells	METTL3; YTHDF1; lnc-Dpf3 remodeling	Antigen-processing transcripts; lnc-Dpf3; mTOR-associated signaling	Antigen presentation, glycolysis and migration	Functional endpoints differ; expression and genetic studies are summarized in Table 1	Genetic or pharmacological perturbation [48,49,50]
NK cells	FTO	SOCS family transcripts	Cytokine signaling and cytotoxicity	NK cell-intrinsic mechanism; direct glycolytic flux not established by this study	Cell experiments and mouse functional evidence [56]
T cells and tumor microenvironment	METTL3, FTO, YTHDF2	Fas, PDK1, ELK3 and others	Survival, tumor glycolysis, immune suppression	Normal T cell, malignant T cell and tumor cell mechanisms are distinct	Model-specific functional evidence [66,68,69,70,71,72,89,90]
CD4^+^ T cells	METTL3, METTL14, FTO, WTAP	SLC2A1, SOCS3, FOXP3, RORA/RORC, SCD1, THBS1, ICOS	Differentiation and model-specific metabolic changes	T cell-intrinsic pathways; metabolic endpoints are not established equally across studies	Perturbation studies; direct metabolism in selected models [19,74,75,76,77,78,79,81]
Macrophage-to-CD4^+^ T cell communication	METTL3; YTHDF3	PGP	PPP-associated metabolism, respiration and Th1 differentiation	Initial perturbation in macrophages; downstream T cell response	Myeloid deletion, macrophage coculture and metabolic measurements [80]
Normal, malignant and autoimmune B cells	FTO, YTHDF2 and others	CPT1A, ATP6V1G1; ATP synthesis and immune recognition transcripts	Development, FAO, mitochondrial function, immune evasion	Developmental evidence is not equivalent to metabolism; YTHDF2 ATP branch involves m5C	Reviews and model-specific mechanisms [22,82,83,84,85,86,87,88]
Microglia and retinal endothelium	FTO, ALKBH5, YTHDF2	cGAS, A20, ATG9A, Mxd1, PARP14; endothelial CDK2	Inflammatory signaling, ferritinophagy, stress responses	Predominantly preclinical microglial models; CDK2 pathway originates in endothelial cells	Cell and animal models; variable metabolic endpoints [94,95,96,97,98,99,100,101,102]
Kupffer cells	METTL3/METTL14	TGF-β1	Inflammatory and fibrotic responses	Kupffer cell mechanism with myeloid genetic validation; direct lipid flux not established	Cellular and mouse mechanism [103]
Macrophages in endometriosis	METTL3; METTL14/YTHDF2	TRIB1; KLF4	Macrophage polarization; lactate-associated effects in the TRIB1 study	Stromal–immune interactions in lesions and coculture; not a general reproductive tissue model	Clinical associations plus cell/animal or coculture experiments [45,46]

**Table 3:** Pharmacological interventions and clinical development relevant to m^6^A-associated immune regulation.

Cellular Context	Intervention	Target Relationship	Reported Endpoint	Disease or Model	References and Evidence
Macrophages	α-KG	Metabolite supporting FTO-associated activity	STAT3 regulation and reparative polarization	Myocardial infarction	Cell/mouse mechanism; not a selective FTO drug [34]
Myeloid/macrophage compartment	STM2457	METTL3 inhibitor	Inflammatory and cardiopulmonary remodeling endpoints	Pulmonary hypertension	Mouse drug/genetic models; specific glycolytic mechanism not established [58]
Liver and macrophage models	STM2457	METTL3 inhibitor	ACSL4-associated ferroptosis, polarization and fibrosis	Alveolar echinococcosis	Preclinical pharmacology; no clinical efficacy established [59]
Tumor cells with CAR-NK therapy	STM2457-loaded hydrogel	Local METTL3 inhibition	Tumor chemokine output, NK recruitment and tumor control	Colorectal cancer	Delivery and combination therapy models [60]
Malignant T cells	Dac51	FTO inhibitor	ELK3-linked glycolysis and leukemia growth	T cell acute lymphoblastic leukemia	Cell and animal studies; not normal T cell metabolism [90]
RA neutrophils and clinical cohort	Xinfeng Capsule	ALKBH5-associated formula; direct binding unverified	ALKBH5/LINC00968, oxidative stress and NETosis	Rheumatoid arthritis	Retrospective cohort plus in vitro rescue; docking only for direct binding [61]
Alloreactive CD4^+^ T cells	STM2457	METTL3 inhibitor	Reduced proliferation and Th1 differentiation	Allogeneic response model	Cellular pharmacology; no direct metabolic flux endpoint [89]
Breast cancer cells	SKLB-Y13	Selective YTHDF1 inhibitor	Reduced PRPF6 translation and tumor growth	Breast cancer	Binding, cellular and animal studies; T cell metabolic efficacy untested [91]
Patients with advanced malignancies	STC-15; alone or with toripalimab	Investigational METTL3 inhibitor	Safety, tolerability and antitumor activity under clinical evaluation	Advanced cancers; selected sarcomas	Phase 1 completed; phase 1b/2 recruiting. No posted registry results as of 15 October 2026 [92,93]
Microglia	Vitamin D	VDR-dependent induction of FTO	Mxd1 pathway and reduced inflammatory activation	LPS-induced neuroinflammation	Cell/mouse mechanism; indirect modulation, no established clinical efficacy [101]

## Data Availability

No new data were created or analyzed in this study. Data sharing is not applicable to this article.

## Abbreviations

AID, activation-induced cytidine deaminase; ALKBH5, AlkB homolog 5; α-KG, α-ketoglutarate; D-2HG, D-2-hydroxyglutarate; DC, dendritic cell; FAO, fatty acid oxidation; FTO, fat mass and obesity-associated protein; HIF-1α, hypoxia-inducible factor 1α; m^6^A, N^6^-methyladenosine; METTL3/14, methyltransferase-like 3/14; NK, natural killer; OXPHOS, oxidative phosphorylation; PPP, pentose phosphate pathway; SIGIRR, single immunoglobulin interleukin-1 receptor-related molecule; TAM, tumor-associated macrophage; Tfh, follicular helper T cell; TME, tumor microenvironment; Treg, regulatory T cell; WTAP, Wilms tumor 1-associated protein; YTHDF, YTH N^6^-methyladenosine RNA-binding protein.
